# Application of Three‐Dimensionally Printed Surgical Guides in Precise Sacral Tumor Excision and Defect Reconstruction

**DOI:** 10.1111/os.70383

**Published:** 2026-07-26

**Authors:** Yansong Liu, Yinghua He, Huanwen Ding, Hu Chen, Zhongbiao Liang, Ningling Xie, Renxuan Tu, Qiang Tu

**Affiliations:** ^1^ The First School of Clinical Medicine Southern Medical University Guangzhou Guangdong China; ^2^ Department of Orthopedics General Hospital of Southern Theater Command Guangzhou Guangdong China; ^3^ School of Medicine South China University of Technology Guangzhou Guangdong China; ^4^ Department of Joint Surgery Guangzhou First People's Hospital Guangzhou Guangdong China; ^5^ Ruijin Hospital, Shanghai Jiaotong University Shanghai China; ^6^ Shenzhen College of International Education Shenzhen Guangdong China

**Keywords:** 3D‐printed guiding templates, computer‐aided design (CAD), lumbopelvic reconstruction, sacral tumor resection, surgical precision

## Abstract

**Objective:**

Precise resection of sacral tumors remains technically demanding due to their deep anatomical location and close proximity to critical neurovascular structures. Conventional freehand techniques often result in suboptimal resection margins, excessive blood loss, and compromised lumbopelvic stability. This study evaluated whether patient‐specific three‐dimensional (3D)‐printed guiding templates improve surgical accuracy and perioperative outcomes in sacral tumor resection and reconstruction.

**Methods:**

Nineteen patients undergoing en bloc sacral tumor resection (S1–S3 involvement) with spinopelvic reconstruction (2006–2020) were retrospectively analyzed. Patients were divided into a 3D‐printing group (*n* = 10) and a conventional freehand group (*n* = 9). In the 3D‐printing group, computer‐aided design and 3D‐printed templates were used for osteotomy, screw placement, and defect reconstruction. Perioperative metrics, surgical accuracy, and complications were compared between groups using Welch's *t*‐test and the Hodges–Lehmann method; oncologic events during follow‐up were recorded descriptively.

**Results:**

The 3D‐printing group demonstrated significantly shorter operative time (456.5 ± 62.36 vs. 574.44 ± 114.58 min, *p* = 0.012), reduced blood loss (4081.40 ± 838.99 vs. 5090.0 ± 1059.67 mL, *p* = 0.034), and fewer fluoroscopic exposures (4.2 ± 0.79 vs. 10.0 ± 1.58, *p* < 0.001) compared with the conventional group. Osteotomy accuracy was also superior in the 3D‐printing group, with significantly lower angular deviation (3.33° ± 0.45° vs. 6.79° ± 2.16°, *p* = 0.0012). Postoperative complication rates were comparable (30% vs. 44.4%, *p* = 0.649), but hospital stay was significantly shorter in the 3D‐printing group (10.7 ± 2.71 vs. 18.11 ± 4.01 days, *p* < 0.001).

**Conclusion:**

Patient‐specific 3D‐printed guiding templates enhance precision in sacral tumor excision and reconstruction, improving surgical efficiency and perioperative safety. This computer‐assisted, template‐guided approach represents a valuable advancement for complex sacral oncologic surgery.

## Graphical abstract

Patient‐specific 3D‐printed guiding templates significantly improve surgical precision and efficiency in complex sacral tumor resection and reconstruction compared to conventional freehand techniques. This computer‐assisted workflow reduces operative time, intraoperative blood loss, and fluoroscopy frequency, providing a safer, highly reproducible strategy for spinal oncology surgery.

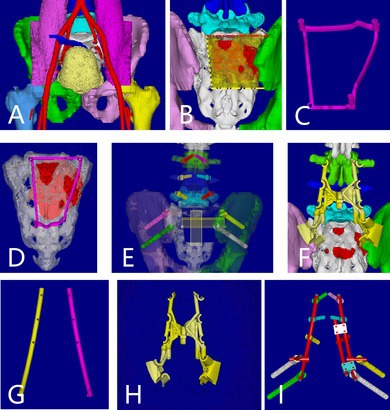

**Abbreviations**
3D: three‐dimensional
ASCF: anterior spinal column fixation
CAD: computer‐aided design
CT: computed tomography
ECT: emission computed tomography
FVF: free vascularized fibular flap
HU: hounsfield units
MRI: magnetic resonance imaging
PET‐CT: positron emission tomography‐CT
SLM: selective laser melting
VAS: visual analog scale

## 1 Introduction

Sacral tumors represent one of the most prevalent categories of spinal neoplasms, primarily comprising primary malignant sacral neoplasms [1]. Owing to their deep anatomical location, these tumors present with subtle and complex clinical manifestations. Due to their insidious onset, sacral neoplasms are often diagnosed at an advanced stage. By the time of diagnosis, these tumors are often large, causing sacral destruction and potentially affecting the sacroiliac joints. Surgical treatment is the definitive approach for the clinical management of sacral tumors [2]. The sacrococcygeal region is highly vascularized and innervated, with a complex anatomical structure adjacent to the presacral venous plexus, pelvic vasculature, cauda equina, rectum, and bladder. Surgical procedures in this region carry a high risk of extensive bleeding, nerve injury, and damage to adjacent organs, potentially resulting in severe complications such as hemorrhagic shock, urinary and fecal incontinence, postoperative wound infections, soft tissue necrosis, and tumor recurrence [3]. Extensive resection of malignant sacral tumors frequently disrupts sacroiliac joint integrity, leading to postoperative lumbopelvic instability [4]. Advanced surgical modalities such as 3D‐printing and computer‐aided design (CAD) have emerged to fulfill the evolving demands for personalized oncology care, demonstrating superior efficacy in complex tumor resections compared to traditional freehand techniques. However, previous clinical research indicates that conventional freehand surgery is inherently limited by the surgeon's subjective visualization and cognitive translation of two‐dimensional imaging into three‐dimensional (3D) anatomy, which frequently leads to inaccurate osteotomy planes, incomplete tumor clearance, or poor implant fitting. Therefore, precise excision of malignant sacral tumors and reconstruction of pelvic ring stability and spinal continuity remain significant surgical challenges, with no established clinical guidelines [5, 6, 7]. To address these critical bottlenecks and bridge the gap between virtual planning and physical execution, this study systematically introduces a patient‐specific, template‐guided surgical framework. Based on our clinical implementation and technical principles, we put forward three core scientific points to guide this research and enrich the reader's understanding: (i) Physical Guidance and Osteotomy Accuracy: To evaluate whether 3D‐printed cutting guides can physically constrain saw orientation, thereby accurately translating CAD‐delineated oncological margins into precise physical bone resections. (ii) Template‐Guided Screw Trajectory and Reconstruction: To analyze the technical application of customized navigation templates in guiding optimal pedicle and iliac screw placement, ensuring stable seating of reconstruction implants and structural continuity.(iii) Surgical Efficiency and Hemorrhage Control: To investigate whether this preplanned, simulation‐driven workflow can minimize intraoperative guesswork and fluoroscopy, synergistically reducing operative time and massive hemorrhage.

## 2 Materials and Methods

### 2.1 General Information

**FIGURE 1:**
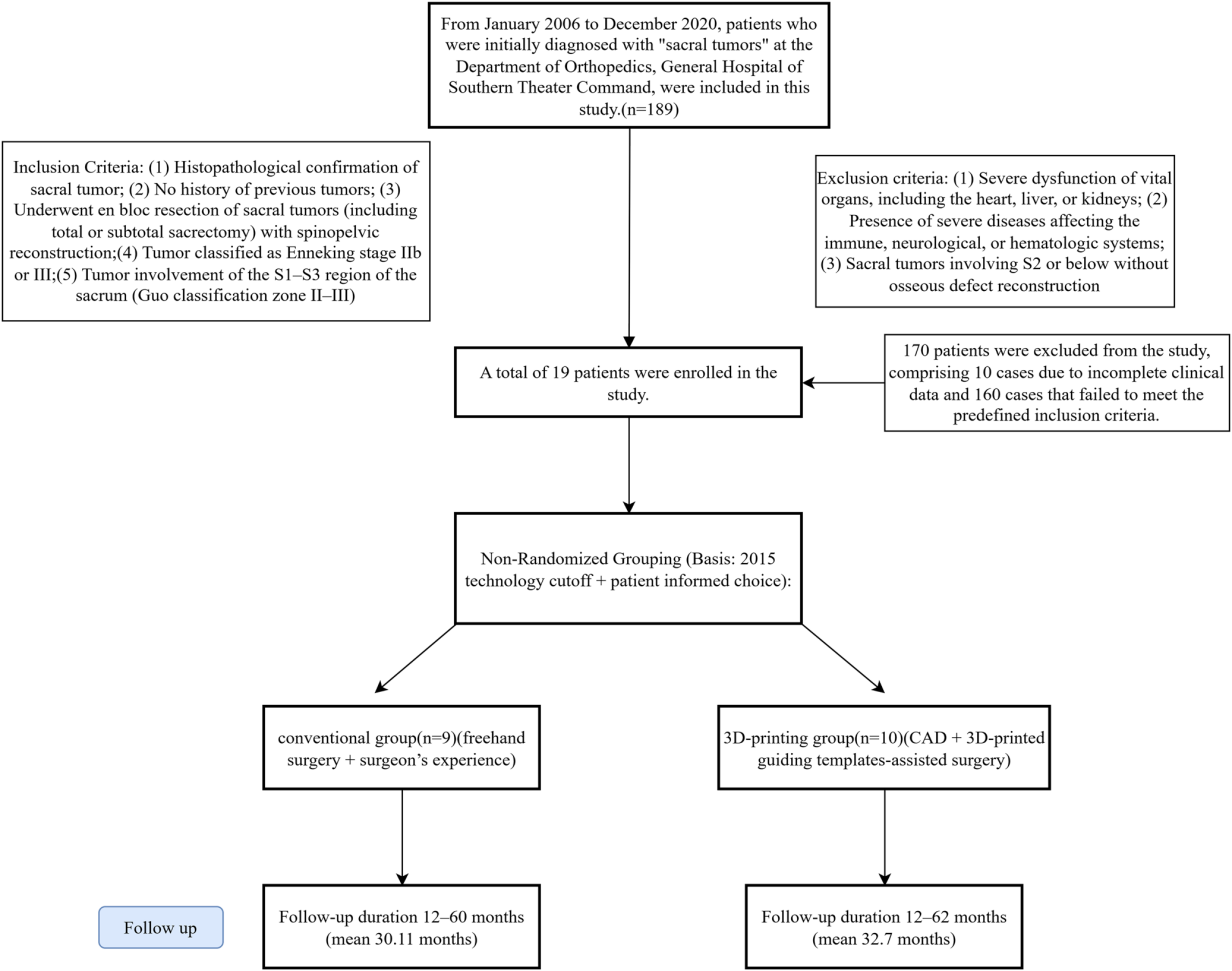
Flowchart of inclusion, exclusion and grouping of patients with sacral tumors (2006–2020).

This single‐center, retrospective cohort study analyzed clinical data from patients who underwent sacral tumor resection with spinopelvic reconstruction at the Department of Orthopaedics, General Hospital of Southern Theater Command, between January 2006 and December 2020. A flowchart summarizing patient screening, inclusion, exclusion, and grouping is presented in Figure 1. A total of 189 patients initially diagnosed with “sacral tumors” during this period were assessed for eligibility. After applying the predefined inclusion and exclusion criteria, 19 patients met all criteria and were finally enrolled. Among them, 10 patients underwent CAD and 3D printing–assisted surgery, while 9 patients received conventional freehand surgery. Following histopathological confirmation, the diagnosis and management strategy for each patient were formulated through a multidisciplinary tumor board (MDT) discussion. The board, comprising orthopedic oncologists, radiologists, pathologists, and radiation oncologists, evaluated surgical feasibility and the potential to achieve tumor‐free margins and stable reconstruction. This ensured that the application of CAD and 3D‐printed guides was grounded in sound oncologic and anatomical rationale. This study received ethical approval from the Ethics Committee of the General Hospital of Southern Theater Command (Approval No. NZLLKZ2025137). The requirement for written informed consent for study participation was waived due to the retrospective use of anonymized data, whereas all patients had providedpreoperatively written informed consent for the surgical procedure.

Patient allocation to the 3D‐printing group (*n* = 10) or the conventional freehand group (*n* = 9) was nonrandomized and evolved with the clinical adoption of 3D‐printing technology at our institution. From 2006 to 2015, when 3D printing was unavailable, all eligible patients were assigned to the conventional group. After 2016, with the technology becoming clinically established, patients were informed of both surgical options—including the potential benefits (e.g., enhanced precision, reduced fluoroscopy) and limitations (e.g., longer preoperative preparation)—and the final approach was determined through shared decision‐making based on informed patient preference. Baseline characteristics were compared between groups, and no significant differences were observed with respect to age, sex, tumor type, or Enneking stage (Table 1), indicating good comparability between cohorts.

**TABLE 1:** Demographic and clinical characteristics of patients.

Variable	3D‐printing group (*n* = 10)	Conventional group (*n* = 9)	*p*
Age (years), mean ± SD	45.2 ± 12.3	48.7 ± 10.9	0.524
Sex (male/female)	6/4	5/4	/
Tumor type			
Osteosarcoma	3 (30%)	2 (22.2%)	/
Sacral mesenchymal chondrosarcoma	1 (10%)	1 (11.1%)	/
Sacral epithelioid hemangioendothelioma	1 (10%)	0	/
Malignant peripheral nerve sheath tumor	1 (10%)	2 (22.2%)	/
Chordoma	1 (10%)	0	
Ewing sarcoma	2 (20%)	3 (33.3%)	/
Giant cell tumor of bone	1 (10%)	1 (11.1%)	
Enneking stage			
Stage IIb	7 (70%)	7 (77.8%)	/
Stage III	3 (30%)	2 (22.2%)	/

### 2.2 Reconstruction Strategy and Chronological Evolution

**FIGURE 2:**
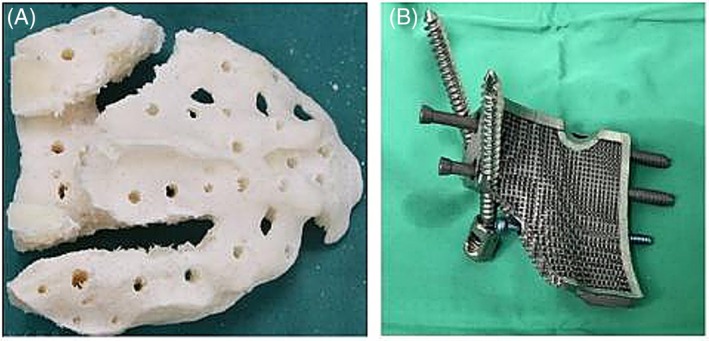
Preoperative preparation of graft and prosthesis for sacral reconstruction. (A) Allografts were trimmed using custom cutting guides to achieve a 3D configuration that precisely matches the post‐resection bony defect. (B) A 3D‐printed personalized sacral prosthesis assembled with screws, prepared for sacral reconstruction.

Over the 15‐year study period (2006–2020), our institutional strategy for sacral defect reconstruction underwent a distinct phased evolution, with patient‐specific 3D‐printed titanium prostheses introduced during the later study period. A total of 15 patients underwent reconstruction using allogeneic bone grafts (Figure 2A), including 8 cases in the 3D‐printing group and 7 cases in the conventional group. The remaining four patients were systematically treated with patient‐specific 3D‐printed titanium prostheses (Figure 2B), with two cases in the 3D‐printing group and 2 cases in the conventional group.

### 2.3 Inclusion and Exclusion Criteria

Following ethical approval, eligible patients were included in the study. The inclusion criteria were (1) histopathological confirmation of sacral tumor; (2) no history of previous tumors; (3) underwent en bloc resection of sacral tumors (including total or subtotal sacrectomy) with spinopelvic reconstruction; (4) tumor classified as Enneking stage IIb or III; (5) tumor involvement of the S1–S3 region of the sacrum (Guo classification zone II–III). The exclusion criteria were (1) severe dysfunction of vital organs, including the heart, liver, or kidneys; (2) presence of severe diseases affecting the immune, neurological, or hematologic systems; (3) sacral tumors involving S2 or below without osseous defect reconstruction.

**FIGURE 3:**
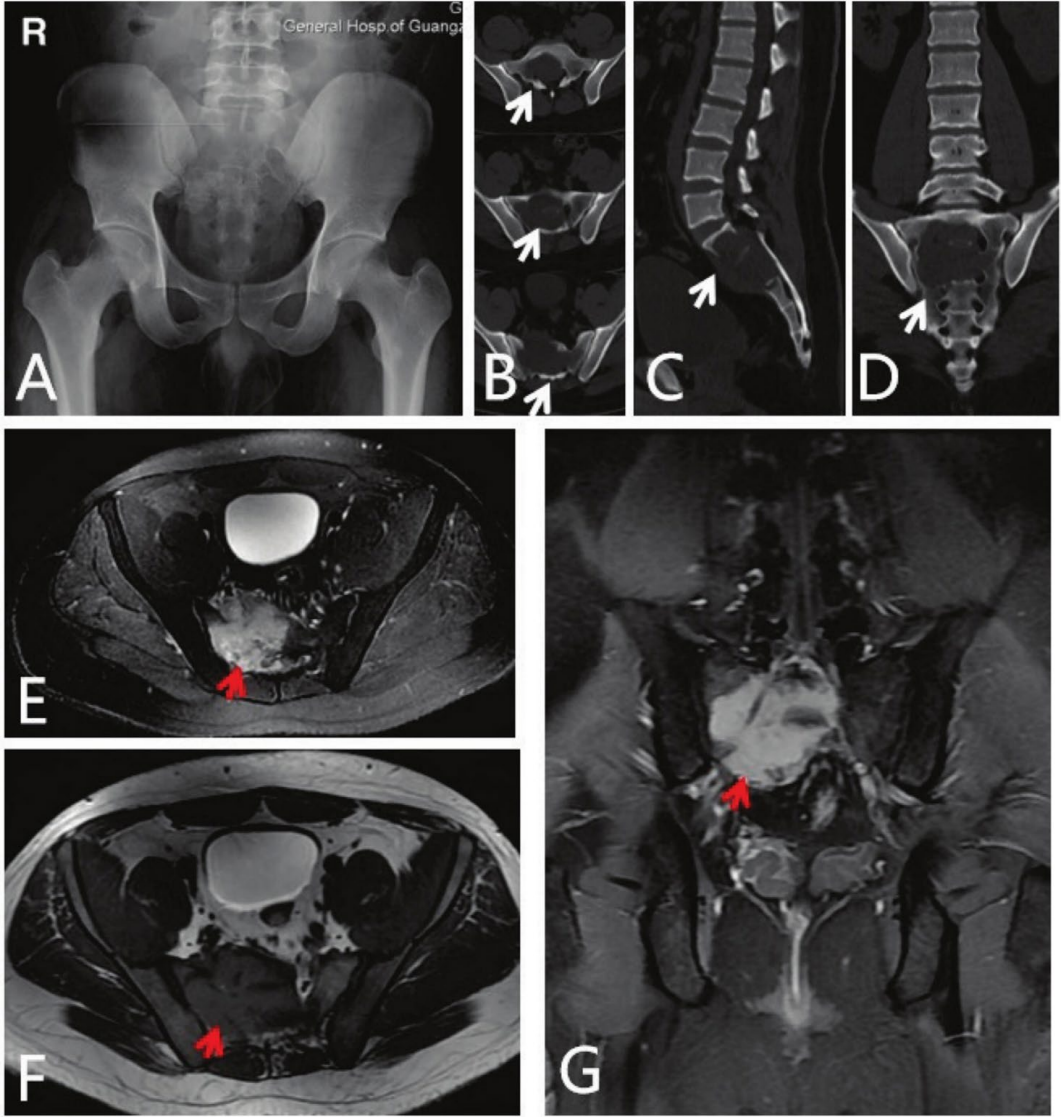
A 24‐year‐old male patient with lumbosacral pain for 2 months and dysuria for 1 month. (A) Preoperative anteroposterior radiograph of the pelvis and (B–D) preoperative CT scans demonstrating bone destruction involving S1, S2, S3 and the right‐sided region, with disappearance of the S1 and S2 anterior sacral foramina. (E–G) Preoperative MRI showing tumor infiltration of S1‐S3, extending rightward to the sacral margin and downward to mid‐S3.

A total of 19 patients were included and divided into two groups based on the treatment approach. All patients underwent preoperative imaging evaluations, including radiography, computed tomography (CT), magnetic resonance imaging (MRI) (Figure 3), and emission computed tomography (ECT) or PET‐CT.

### 2.4 Construction of 3D Models and Preoperative Planning

**FIGURE 4:**
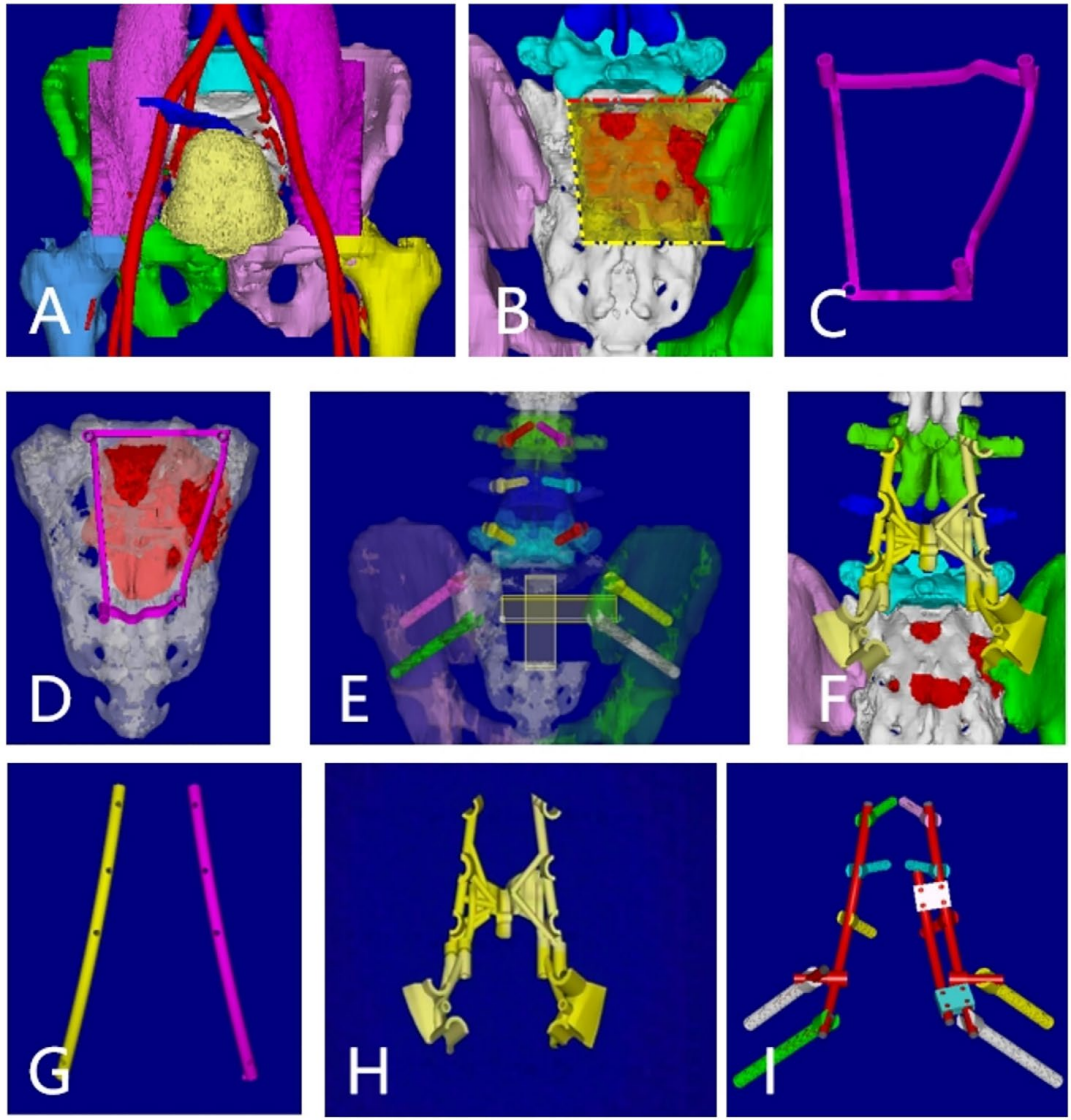
Preoperative planning: (A) Three‐dimensional CT reconstruction: visualization of the spatial relationship between the sacral tumor and critical anatomical structures, including the bilateral internal and external iliac vessels, bladder, and rectum. (B) Assessment of tumor invasion extent and simulated resection margins derived from CT‐MRI image fusion. (C) CAD model of a personalized sacral osteotomy guide template designed for precise resection of the sacral defect. (D) 3D reconstructed views showing the simulated placement of the osteotomy guide template on the sacrum, confirming its anatomic conformity. The osteotomy guide includes a patient‐matched contact surface, planar cutting faces (guiding planes), and pinholes for temporary K‐wire fixation. (E) Preoperative 3D virtual planning: simulation of pedicle‐iliac screw insertion to optimize trajectory and angulation. (F) CAD‐based simulation: overlay of the pedicle‐iliac screw drill‐guiding templates onto 3D models of the lumbar spine and sacrum to verify positional accuracy. (G) Pre‐bent connecting rods selected according to the planned screw positions and spinal morphology. (H) CAD design of the pedicle–iliac screw drill‐guiding templates. (I) Overall system integration: full‐scale assembly of the pedicle‐iliac screw internal fixation system featuring a left single‐rod and right double‐rod configuration to minimize the risk of rod fracture during sacral defect reconstruction.

All patients with malignant bone tumors underwent X‐rays, CT, MRI, and ECT/PET‐CT to assess for metastasis, with neoadjuvant chemotherapy added for osteosarcoma/Ewing sarcoma. For the 3D‐printing group, DICOM‐format CT/MRI data were stored and processed: CT (L3 to greater trochanters) captured bony/tumor structures, while MRI detailed soft tissue infiltration. These datasets were imported into Mimics software (Materialize, Leuven, Belgium) for 3D reconstruction of the sacrum, pelvis, lumbar spine, sacroiliac joints, and tumor margins—bony segmentation used Hounsfield Units (HU) thresholding/region‐growing, with manual correction of blurred boundaries (Figure 4).

Imageware software (UGS Corp., Plano, Texas, USA) was used to measure specific parameters critical for surgery: these included (1) tumor dimensions; (2) distances from tumor margins to key neurovascular structures (e.g., S1–S3 nerve roots, internal iliac vessels); (3) optimal trajectories and lengths for the implantation of pedicle screws and iliac screws.

Preoperative planning integrated repeated simulation of resection, reconstruction, and screw placement validation. Surgical margins were delineated according to the Enneking staging system and Guo's classification for sacral tumors [8, 9]. Malignant tumors required excision margins extending 3–5 cm beyond the tumor infiltration area to include adjacent normal bone and 1–2 cm of surrounding soft tissue. These adjustments informed the design of resection boundaries, ensuring the osteotomy template would accommodate subsequent reconstruction. Allogeneic grafts/3D‐printed prostheses were tested in the defect, with dimension adjustments for lumbar/pelvic alignment. Guided by these simulations and predefined margins, a patient‐specific osteotomy guide template was first designed using CAD software (UG‐NX6) and reverse engineering principles to facilitate precise resection (Figure 4C,D). The guiding planes functioned as planar cutting faces to constrain saw orientation during osteotomy, whereas the pinholes enabled temporary K‐wire fixation to resist rotational or translational migration. Points, lines, and surfaces extracted from the anatomical model were used to develop this tumor resection template, with individualized guides manufactured via 3D printing using photosensitive resin—ensuring alignment with preplanned resection boundaries. Following confirmation of optimal resection boundaries via the osteotomy template, attention turned to stabilizing the residual lumbopelvic structure through precise screw placement, supported by customized navigation templates. Through repeated simulation, optimal pedicle and iliac screw trajectories and lengths were first validated and confirmed in the reconstructed 3D model (Figure 4E). Based on these predetermined trajectories and integrated with bony anatomical features, the pedicle and iliac screw navigation templates were then iteratively designed (Figure 4F,H)—this refined design process ensured accurate screw placement by aligning with bony landmarks while avoiding neurovascular structures. Parameters such as entry points, angles, and screw lengths were finalized based on these validated outcomes. For subsequent reconstruction, according to the spinal morphology and the planned positions of pedicle/iliac screws, the pre‐bent curvature and length of the connecting rod were selected to fit anatomical characteristics (Figure 4G). These rods, combined with the pre‐placed screws, formed a stable internal fixation system to restore lumbopelvic stability (Figure 4I).

Following completion of virtual planning and template design, the patient‐specific guides were fabricated preoperatively using photosensitive resin and underwent routine post‐processing and sterilization before surgery. The typical turnaround time from image transfer to guide availability was approximately 1 week, and the estimated direct cost for one set of guides was approximately 6000 RMB per patient, excluding personnel‐related costs and routine operating room expenses.

### 2.5 Surgical Procedure

Given the anatomical complexity and extent of sacral involvement in this cohort, all patients were treated using an en bloc resection framework (total or subtotal sacrectomy) with spinopelvic reconstruction. Resection margins were planned preoperatively according to oncologic principles and individualized based on tumor extent and anatomical constraints, with the intent to achieve margin‐negative en bloc resection whenever feasible. The extent of sacral nerve root management and any adjacent visceral resection was determined by tumor involvement and oncologic safety, rather than by template use itself. Under general anesthesia, most patients underwent a posterior approach alone in a prone position, received tumor embolization within 24 h prior to surgery to minimize blood loss, and underwent standard preoperative preparations including blood preparation (3000–5000 mL), bowel cleansing, and urinary catheterization. A posterior median incision was made, tailored as an inverted “Y”‐shape for large or bilateral lesions or a curved incision for smaller or unilateral tumors, to ensure sufficient surgical exposure. For complex cases with anterior invasion, a combined anterior–posterior approach was adopted.

In the conventional group, tumor resection and reconstruction were performed freehand. Pedicle screw‐rod systems were placed based on anatomical landmarks identified intraoperatively, with resection margins determined by preoperative imaging data (CT/MRI) and the surgeon's clinical experience. Intraoperative fluoroscopy was repeatedly used to assist in tumor localization and defect reconstruction, though precise resection boundaries relied entirely on manual judgment. For defect reconstruction, implants (allogeneic bone grafts or 3D‐printed prostheses) were utilized according to the individualized reconstruction plan. In cases reconstructed with allografts, the grafts were trimmed intraoperatively by hand to approximate the defect shape, guided by the surgeon's visual assessment and tactile feedback to achieve anatomical alignment, then secured with locking plates and screws. In cases reconstructed with 3D‐printed prostheses, the implants were prefabricated via CAD and rapid prototyping to match the defect anatomy, but their intraoperative placement was adjusted manually rather than via template guidance. Regardless of the reconstruction material, lumbar and iliac screw‐rod systems were applied to restore pelvic stability, and preserved muscles were reattached to the graft/prosthesis or adjacent bones.

However, the surgical procedure in the 3D‐printing group differed. Following meticulous blunt dissection of the soft tissues while maintaining the structural integrity of the osseous and ligamentous components, the intended bony contact surface was fully exposed. Residual soft tissue remnants on the seating area were meticulously debrided to ensure precise template registration. Preoperatively, the CAD‐derived positioning markers were identified, and the osteotomy guiding template was securely positioned against the posterior sacral osseous landmarks to achieve stable and flush alignment. Proper seating was verified by the absence of visible gaps and through manual stability assessment. The guiding template was subsequently affixed to the osseous landmarks using Kirschner wires to prevent intraoperative displacement. Osteotomies were performed by advancing an oscillating saw along the predefined guiding planes of the template, with deeper sections and angular regions completed using osteotomes as necessary.

**FIGURE 5:**
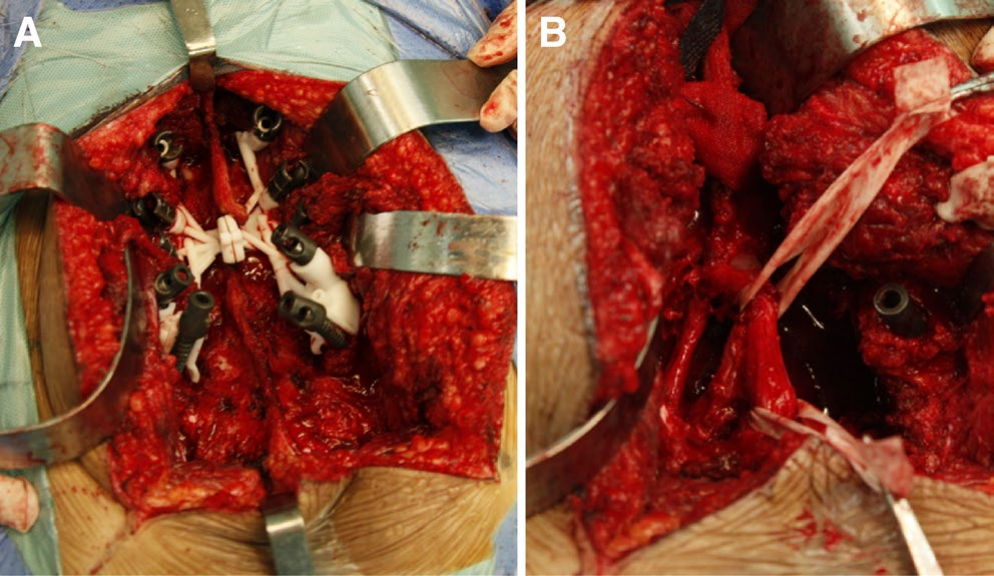
Intraoperative view. (A) Pedicle‐iliac screw placement template utilized for screw placement; (B) resection of posterior tumor tissue.

**FIGURE 6:**
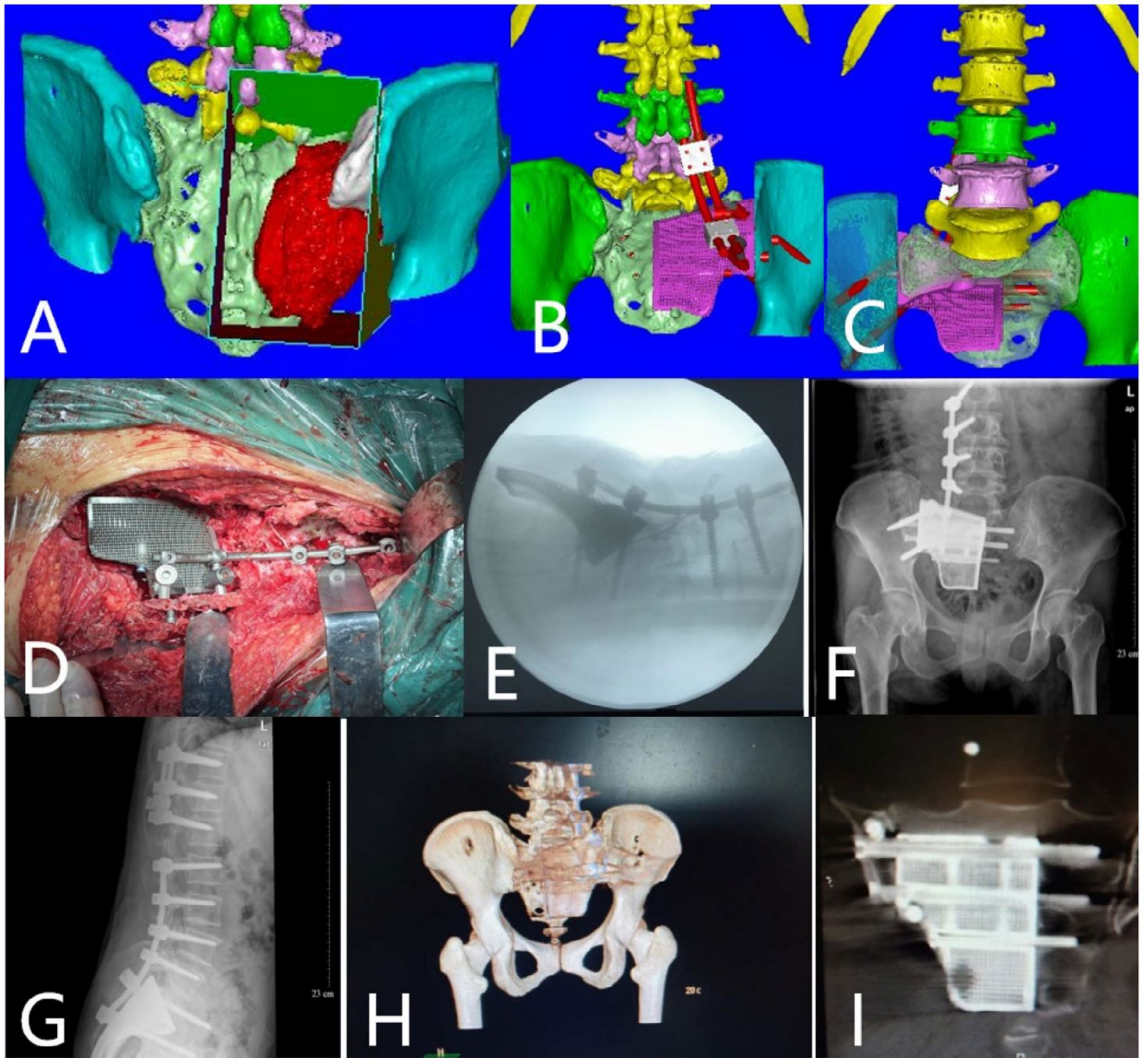
A 52‐year‐old female patient with right lower limb pain and movement limitation for over 16 months, diagnosed with chordoma. (A) Three‐dimensional CT reconstruction combined with MRI fusion illustrating tumor extent and simulated resection margins in spatial relation to the sacrum. (B, C) Preoperative 3D virtual planning and CAD‐based simulation of sacral defect reconstruction using a patient‐specific prosthesis combined with a pedicle–iliac screw internal fixation system, optimizing implant position and construct configuration. (D) Intraoperative view showing placement of the 3D‐printed prosthesis following guide‐assisted osteotomy. (E) Intraoperative C‐arm fluoroscopy confirming final positioning of the prosthesis and screws. (F, G) Postoperative anteroposterior and lateral radiographs demonstrating stable fixation. (H, I) Postoperative CT three‐dimensional reconstructions illustrating complete tumor resection and reconstructed sacral contour.

Soft‐tissue resection was not restricted by the surgical template and was conducted based on the preoperative MRI‐delineated tumor infiltration boundaries and intraoperative oncological assessment, with the objective of attaining the planned 1–2 cm soft‐tissue margin where anatomically permissible. Following excision, the surgical field was irrigated with distilled water for 15 min as part of routine wound irrigation per institutional protocol. For defect reconstruction, the respective implants were positioned using preoperative templates: allografts were trimmed via 3D‐printed guiding templates to achieve anatomical fit, and prostheses were implanted according to the preplanned reconstruction geometry. Both allografts and prostheses were then secured through lumbar and iliac screw placement, with screw trajectories precisely guided by 3D‐printed navigation templates (Figure 5); final internal fixation was performed to ensure stable integration with the lumbopelvic complex. Intraoperative placement of the 3D‐printed titanium prosthesis was performed according to the preplanned trajectory, with the prosthesis closely matching the defect morphology (Figure 6D). The interface between the prosthesis and host bone was precisely aligned under the guidance of templates, and screw fixation was completed to achieve immediate stability (Figure 6E). Intraoperative fluoroscopy was used only to confirm optimal positioning of the prosthesis and screws. Following thorough hemostasis and irrigation, drainage tubes were placed, and the incision was closed in layers.

### 2.6 Postoperative Management

Postoperatively, prophylactic antibiotics were routinely administered to prevent wound infection. Surgical drains were maintained and removed only when the drainage output decreased to < 50 mL/day. Patients were encouraged to begin early ankle and knee range‐of‐motion exercises, followed by gradual assisted mobilization as tolerated. Postoperative chemotherapy, when indicated, was initiated based on wound healing status and multidisciplinary recommendations from the oncology team. In this retrospective cohort, discharge timing was determined according to routine clinical practice; however, discharge was generally considered once patients achieved stable vital signs, adequate pain control with oral analgesics, no complications requiring ongoing inpatient management (e.g., uncontrolled infection or wound issues), and the ability to mobilize with assistance. According to our institutional guidelines, patients in this cohort were highly encouraged to be systematically monitored in our outpatient clinic at recommended chronological intervals: once every 3 months during the first postoperative year, every 6 months during the second and third years, and annually thereafter, with pragmatic, compliant scheduling deviations allowed based on the patient's geographic and functional status. Follow‐up assessments included regular radiographs and CT to monitor implant position, loosening, fracture, and other reconstruction‐related complications (Figure 6F–I). For patients reconstructed with massive allografts, the graft–host junction was evaluated for evidence of osseous union. For those receiving patient‐specific 3D‐printed prostheses, integration was assessed by examining bone ingrowth at the bone–implant interface.

### 2.7 Assessment of Osteotomy Accuracy

For accuracy analysis, postoperative CT datasets were reconstructed in Mimics software (Materialize, Leuven, Belgium) and reconstructed into 3D bone models. The preoperative planned osteotomy planes derived from CAD simulations were likewise reconstructed and spatially aligned with postoperative CT models based on stable anatomical landmarks of the residual sacrum and pelvis.

For each osteotomy plane, a best‐fit geometric plane was generated using surface point sampling in Mimics. The orientation of each plane was represented by its normal vector. Angular deviation was calculated as the 3D angle between the normal vectors of the planned osteotomy plane and the corresponding postoperative osteotomy surface, providing a quantitative indicator of consistency between preoperative planning and intraoperative execution.

Millimeter‐level positional deviation or surface offset analysis was not performed due to significant metal artifacts from extensive spinopelvic fixation constructs, which obscured precise visualization of resection boundaries and bone–implant interfaces on postoperative CT images.

To enhance measurement reliability, each angular measurement was performed independently by two experienced observers, and the mean value was used for final analysis.

### 2.8 Outcome Measures

The primary radiographic outcome was osteotomy accuracy, defined as the angular deviation between the preoperatively planned osteotomy plane and the corresponding postoperative osteotomy surface. Smaller angular deviations indicated greater consistency between virtual planning and actual surgical execution.

Secondary outcome measures included operative time, intraoperative blood loss, length of hospital stay, fluoroscopy frequency, postoperative complications, and pain intensity. Operative time was defined as the duration from skin incision to wound closure. Intraoperative blood loss was obtained from anesthetic records. Fluoroscopy frequency was recorded as the total number of intraoperative fluoroscopic acquisitions. Pain intensity was assessed using the visual analog scale (VAS) preoperatively, on the operation day, and on postoperative Day 7. Postoperative complications were assessed through clinical records during follow‐up.

For osteotomy accuracy assessment, angular deviation was measured using the CT‐based co‐registration and 3D plane analysis method described above. Measurements were independently performed by two experienced observers, and the mean value was used for final analysis. Millimeter‐level positional deviation was not assessed because metal artifacts from extensive spinopelvic fixation prevented reliable delineation of osteotomy margins on postoperative CT images.

### 2.9 Statistical Analysis

All data were analyzed using SPSS version 21.0 (IBM SPSS, USA). Continuous variables were expressed as mean ± standard deviation, and categorical variables as counts and percentages. For continuous variables with unequal variances (e.g., operative time and blood loss), Welch's *t*‐test was applied, and a two‐tailed *p* value < 0.05 was considered statistically significant. For nonparametric comparisons, the median difference and corresponding 95% confidence interval were estimated using the Hodges–Lehmann method to enhance result interpretability.

## 3 Results

### 3.1 Basic Information

From January 2006 to December 2020, a total of 19 patients (11 males) who underwent sacral tumor resection and reconstruction were included in the study, with 10 patients in the 3D‐printing group and 9 patients in the conventional group. The mean age of the patients was 45.2 ± 12.3 years in the 3D‐printing group and 48.7 ± 10.9 years in the conventional group (*p* = 0.524). The average follow‐up duration was 30.11 months (range, 12–60 months) in the conventional group and 32.7 months (range, 12–62 months) in the 3D‐printing group. The demographic and clinical characteristics of both groups are summarized in Table 1. Pathological diagnoses included osteosarcoma (*n* = 5), Ewing sarcoma (*n* = 5), malignant peripheral nerve sheath tumor (*n* = 3), mesenchymal chondrosarcoma (*n* = 2), epithelioid hemangioendothelioma (*n* = 1), giant cell tumor of bone (*n* = 2), and chordoma (*n* = 1). All the variables mentioned earlier were not significantly different between the 2 groups.

### 3.2 Surgical Outcomes

#### 3.2.1 Intraoperative Efficiency and Osteotomy Precision

The 3D‐printing group demonstrated significantly superior surgical efficiency and precision compared to the conventional group. The mean operative time in the 3D‐printing group was significantly shorter than that in the conventional group (456.50 ± 62.36 vs. 574.44 ± 114.58 min, *p* = 0.012). Similarly, intraoperative blood loss was significantly reduced (4081 ± 839 vs. 5090 ± 1060 mL, *p* = 0.034). Notably, the 3D‐printing group required substantially fewer intraoperative fluoroscopic views (4.2 ± 0.79 vs. 10.0 ± 1.58, *p* < 0.001).

Regarding osteotomy precision, the mean angular deviation between the postoperative CT scans and preoperative planes was 3.33° ± 0.45° in the 3D‐printing group, which was significantly lower than the 6.79° ± 2.16° observed in the conventional group (*p* = 0.0012). While significant metallic artifacts from internal fixation precluded millimeter‐level positional assessment of the bone–implant interface, qualitative assessment and negative histopathological margins confirmed the adequacy of tumor resection and implant placement consistency (Figure 6).

#### 3.2.2 Clinical Follow‐Up Observations

Detailed individual patient data, including specific tumor pathology, resection and reconstruction methods, functional status, and follow‐up outcomes, are comprehensively summarized in Table 2. Patients in the 3D‐printing group were followed up for 12 to 62 months, with an average duration of 32.7 months. Detailed observations during follow‐up were as follows: (1) One patient with osteosarcoma (Enneking IIb) developed rapid metastatic progression to the lungs and liver 10 months postoperatively, presenting with intractable pain and respiratory failure; the patient died 12 months after surgery, resulting in premature termination of follow‐up. (2) None developed rectovesical fistula or hemorrhagic shock. (3) Nine patients were able to ambulate normally, while one required a walker due to numbness in the soles of the feet. (4) Urinary and bowel functions: Eight patients regained normal urinary and bowel function within 3 months postoperatively; one patient experienced normal urinary function but had poor anal sphincter reflexes requiring frequent laxatives or suppositories; one patient developed urinary and bowel dysfunction 2 months postoperatively. (5) Other complications: Two patients developed postoperative wound infections, which were effectively managed with anti‐inflammatory medications, debridement, suturing, and tube irrigation. One patient sustained a fall, resulting in an internal fixation rod fracture, necessitating a second surgery for replacement. Additionally, one case of sacral chordoma recurred 3 years postoperatively.

**TABLE 2:** Detailed clinical characteristics, surgical procedures, and follow‐up outcomes of all 19 patients.

No.	Group	Age	Sex	Diagnosis	Enneking stage	Postoperative complications	Functional status (ambulation/bowel/urinary)	Sacral level involved	Resection type	Reconstruction	Recurrence/metastasis (months)	Final disease status	Follow‐up period (months)
1	3D	24	M	Giant cell tumor of bone	IIb	None	Normal	S1–S3	En bloc	Allograft		NED	42
2	3D	30	M	Osteosarcoma	IIb	Wound infection	Normal	S1–S3	En bloc	Allograft		NED	24
3	3D	34	M	Osteosarcoma	IIb	None	Normal	S1–S3	En bloc	Allograft		NED	18
4	3D	47	F	Osteosarcoma	IIb	None	Normal	S1–S3	En bloc	Allograft	10	DOD	12
5	3D	47	F	Sacral mesenchymal chondrosarcoma	IIb	None	Plantar numbness, requires walker	S1–S3	En bloc	Allograft		NED	38
6	3D	48	M	Sacral epithelioid hemangioendothelioma	III	Wound infection	Normal	S1–S3	En bloc	3D‐printed prosthesis		NED	62
7	3D	49	M	Malignant peripheral nerve sheath tumor	IIb	None	Urinary/bowel dysfunction	S1–S3	En bloc	Allograft		NED	35
8	3D	52	F	Chordoma	IIb	None	Normal	S1–S3	En bloc	3D‐printed prosthesis	36	AWD	48
9	3D	58	F	Ewing sarcoma	IIb	None	Normal	S1–S3	En bloc	Allograft		NED	30
10	3D	63	M	Ewing sarcoma	IIb	Internal fixation rod fracture (due to fall)	Poor anal sphincter reflex	S1–S3	En bloc	Allograft		NED	18
11	Conventional	33	M	Osteosarcoma	IIb	None	Normal	S1–S3	En bloc	Allograft	15	DOD	18
12	Conventional	39	F	Osteosarcoma	IIb	Wound healing complication; internal fixation rod fracture	Normal	S1–S3	En bloc	Allograft		NED	45
13	Conventional	39	M	Sacral mesenchymal chondrosarcoma	IIb	None	Urinary dysfunction	S1–S3	En bloc	3D‐printed prosthesis		NED	15
14	Conventional	48	M	Malignant peripheral nerve sheath tumor	IIb	Wound healing complication	Plantar numbness	S1–S3	En bloc	3D‐printed prosthesis		NED	36
15	Conventional	49	F	Malignant peripheral nerve sheath tumor	IIb	None	Normal	S1–S3	En bloc	Allograft		NED	60
16	Conventional	49	M	Ewing sarcoma	IIb	None	Urinary dysfunction	S1–S3	En bloc	Allograft	3	AWD	26
17	Conventional	52	M	Ewing sarcoma	IIb	None	Normal	S1–S3	En bloc	Allograft	6	AWD	32
18	Conventional	63	F	Ewing sarcoma	III	None	Normal	S1–S3	En bloc	Allograft	9	DOD	12
19	Conventional	66	F	Giant cell tumor of bone	IIb	None	Plantar numbness	S1–S3	En bloc	Allograft		NED	27

Abbreviations: AWD = alive with disease; DOD = dead of disease; NED = no evidence of disease.

Patients in the conventional group were followed up for 12–60 months, with an average follow‐up duration of 30.11 months. None of the nine patients experienced severe perioperative complications. The detailed follow‐up findings were as follows: (1) One patient with Ewing sarcoma (Enneking III) developed widespread bone metastases 9 months postoperatively, leading to pathological fractures and multiorgan failure, and died 12 months after surgery; another patient with osteosarcoma (Enneking IIb) experienced local tumor recurrence with invasion of the presacral plexus and rectum 15 months postoperatively, progressing to cachexia and dying 18 months after surgery. Both cases died during follow‐up. (2) Seven patients regained normal ambulation, while two developed plantar numbness. (3) Seven patients gradually regained urinary and bowel function within 3 months postoperatively, while two developed urinary dysfunction 1 month postoperatively. (4) Local recurrence occurred in two additional patients. One patient with sacral Ewing sarcoma experienced recurrence in the right ilium 6 months postoperatively, requiring wide resection. Another patient with sacral Ewing sarcoma developed recurrence in the presacral soft tissue 3 months postoperatively. (5) Two patients developed wound healing complications, necessitating debridement and secondary wound closure. Additionally, one of these patients experienced an internal fixation rod fracture without obvious trauma, requiring a second surgery for replacement.

Regarding postoperative pain assessment, the 3D‐printing group showed a VAS score of 6.10 ± 1.20 on the operation day and 2.00 ± 0.82 on the seventh postoperative day, while the conventional group had corresponding scores of 6.22 ± 0.97 and 2.22 ± 0.67. There were no statistically significant differences in pain scores between the two groups (both *p* > 0.05, Table 3).

**TABLE 3:** Comparison of surgical outcomes for all patients included in this study.

Variable	3D‐printing group (*n* = 10)	Conventional group (*n* = 9)	*p*
Total operative time (min)	456.50 ± 62.36	574.44 ± 114.58	0.012
Blood loss (mL)	4081.40 ± 838.99	5090.00 ± 1059.67	0.034
Hospital stay (days)	10.7 ± 2.71	18.11 ± 4.01	< 0.001
VAS (op day)	6.10 ± 1.20	6.22 ± 0.97	0.811
VAS (post‐op D7)	2.00 ± 0.82	2.22 ± 0.67	0.528
Postoperative complications (%)	30 (3/10)	44.4 (4/9)	0.649
Mean angle deviation of osteotomy	3.33 ± 0.447	6.79 ± 2.16	0.0012
Mean times for fluoroscopy (number of times)	4.2 ± 0.79	10.0 ± 1.58	< 0.001

Abbreviations: Op day = operation day, Post‐Op D7 = the 7th postoperative day, VAS = visual analog scale.

## 4 Discussion

Sacral tumors present a complex surgical challenge. Due to the capacity of the sacral canal and pelvic cavity to accommodate regional expansion, sacrococcygeal tumors are often large by the time they are clinically detected. Additionally, the sacrum is located deep within the pelvis and is adjacent to critical neurovascular structures and pelvic organs. Its complex bony anatomy—such as the sacroiliac joint and overhanging iliac crest—further complicates surgical exposure and complete resection.

Traditional surgical approaches (e.g., anterior, posterior, or combined approaches) are frequently limited by the tumor's size and anatomical constraints, making precise resection difficult and increasing the risk of residual tumor. To address this critical challenge, we employed 3D‐printed guiding templates combined with preoperative precision planning and intraoperative navigation to achieve accurate tumor resection and personalized reconstruction. This technique not only improves the reproducibility of planned osteotomy planes but also maximizes the preservation of neurological function, thereby reducing the risk of postoperative impairments in ambulation, urination, and defecation.

### 4.1 Role of CAD and 3D‐Printed Guides in Surgical Margin Delineation

Conventional sacral tumor surgery relies heavily on the surgeon's ability to mentally reconstruct 3D anatomy from two‐dimensional imaging, which inherently introduces cognitive limitations. A study by Yonemoto et al. [10] demonstrated that even with multiplanar reconstruction using MRI/CT, intraoperative misjudgment of gluteal muscle complex invasion at the posterior margin can lead to local recurrence rates as high as 75%. This high error rate arises from two critical factors: (1) Imaging limitations: CT is sensitive to bone destruction but has poor soft tissue resolution, while MRI can show tumor infiltration but lacks precise localization of bony landmarks. (2) Surgical execution: Freehand techniques cannot accurately translate preoperative planning into 3D osteotomy planes. Such limitations may lead to variability in intraoperative margin interpretation and osteotomy execution. The present investigation addressed these challenges through two key technological advances: Precision Mapping via CT‐MRI Fusion: By registering stable bony landmarks as fiducial points, we achieved accurate superimposition of MRI‐derived soft tissue signals onto CT bony structures (Figure 3). This approach significantly minimized spatial uncertainty in tumor margin delineation when compared to conventional treatment planning methodologies. Anatomically Adapted Safety Margins: Utilizing UG‐NX6's surface extension algorithm, Enneking‐recommended 3–5 cm safety margins were transformed into patient‐specific cutting surfaces that conformed to individual pelvic anatomy. This eliminated geometric mismatches inherent in standardized osteotomy protocols.

Notably, the timing of local recurrence differed between the groups: the single local recurrence in the 3D‐printing group occurred at 36 months, whereas the three local recurrences in the conventional group were detected at 3, 6, and 15 months postoperatively. Separately, our results support that patient‐specific templates can help execute preplanned osteotomy planes more consistently in complex sacral resections. Consistent with our findings, Shi et al. [11] validated that 3D‐printed patient‐specific surgical jigs achieved high osteotomy accuracy in total sacrectomy, with a mean angular deviation of 4.27°and position deviation of 4.00 mm, confirming that such templates could effectively translate preoperative planning into precise intraoperative execution.

### 4.2 Surgical Simulation and the Application of 3D‐Printed Guiding Templates in Reconstruction

Extensive bone resection, including sacroiliac joint removal, is often required during sacrectomy for malignant sacral tumors, which compromises the stability of the pelvic ring. Gunterberg et al. conducted an experimental study demonstrating that resection below the S1 segment reduces pelvic ring strength by 30%, whereas resection 1 cm below the sacral promontory results in a 50% reduction in strength [12]. Further research by Hugate et al. [13] reported that sacral resection below the S1 foramen allows patients to bear weight postoperatively without a high risk of fracture, while resections above this level significantly increase the risk of sacral fractures. Similarly, Luo et al. [14] confirmed that higher sacral resection planes gradually decrease pelvic ring stability, with resection involving the S1 vertebral body notably increasing the risk of sacroiliac joint and sacral fractures. Therefore, reconstruction is essential following resections involving S1 and S2, whereas sacral resections below S1 do not always require lumbosacral fixation.

Massive bone defects following sacral tumor resection present considerable reconstructive challenges. Autografts, allografts, and prosthetic implants are widely used. For example, free vascularized fibular flaps (FVFs), when feasible, offer excellent biological integration due to their intrinsic blood supply, accelerating bone healing. Compared with nonvascularized fibular struts, FVFs achieve bone healing in approximately 8 months rather than 12, with a significantly lower risk of nonunion (odds ratio = 7.464, *p* = 0.007) [15]. However, a major drawback of this technique is donor site morbidity.

In earlier cases, we employed size‐matched allogeneic sacral grafts for defect reconstruction. These were CT‐scanned to generate 3D anatomical models, and CAD software was used to simulate the graft implantation process. This approach allowed for precise preoperative trimming of the allograft to fit the defect and ensured optimal integration with internal fixation devices. Furthermore, ligament and muscle reattachment to the allograft helped restore partial load‐bearing capacity and pelvic ring stability. These allografts, being anatomically matched, provide reliable initial mechanical stability without requiring donor site surgery and offer support for pelvic organs (e.g., prevention of rectal prolapse). However, they may provoke immune responses, require prolonged wound drainage, and carry a higher risk of infection. Their long‐term durability under high‐stress conditions remains suboptimal—this limitation is further supported by relevant studies: Goodwin et al. [16] pointed out that fresh‐frozen massive allografts employed in patients with tumors are linked to notably high infection rates, typically around 12%. This data, derived from a large retrospective analysis of tumor‐related allograft applications, aligns with the inherent risks of massive allografts—including immunological mismatch and structural degradation from perioperative radiation—which are also relevant to sacral reconstruction. Notably, pelvic and sacral allograft procedures may carry even higher infection risks due to deep anatomical locations, adjacent visceral structures, and prolonged operative times. Clark et al. [17] further confirmed via cadaveric biomechanical testing that femur strut allografts (FSAs)—a common form of massive allograft—exhibited early fatigue failure (average 856 cycles) under gait‐simulating loading, far inferior to implant‐based reconstruction in long‐term stability.

To overcome these limitations, we later adopted patient‐specific 3D‐printed titanium prostheses. These custom implants eliminate donor site morbidity, closely match the defect morphology, and provide excellent immediate mechanical stability. While our study did not perform direct biomechanical testing—a challenge in clinical human studies due to ethical and practical constraints—the advantages of 3D‐printed prostheses over allografts are corroborated by the above literature. Compared with the 12% infection rate of massive fresh‐frozen allografts in tumor patients reported by Goodwin et al. [16], 3D‐printed titanium prosthesessignificantly reduce immunological rejection risks and associated infection vulnerabilities. Consistent with Clark et al. [17], who found expandable cage (EC) constructs had no fatigue failure over 250,000 cycles, our 3D‐printed prostheses, with personalized load‐bearing design, similarly demonstrated satisfactory mid‐term stability in our cohort, with no implant migration or stress‐related failure observed during follow‐up. The only internal fixation rod fracture in the 3D‐printing group occurred strictly due to an accidental fall. In contrast, the conventional group (using allografts) had 1 case of internal fixation rod fracture without obvious trauma—potentially linked to allograft's poor fatigue resistance highlighted in Clark's biomechanical data. Although fibrous encapsulation may hinder long‐term osseointegration, emerging strategies such as bioactive surface coatings offer promise in enhancing bone‐implant integration and reducing complication rates [18]. In a series of 14 pelvic reconstructions using 3D‐printed implants, wound dehiscence occurred in 35.7% of cases, with only one implant removal due to infection [18]. Similarly, Lv et al. [19] reported that a suspended, modular 3D‐printed sacral implant facilitated spinopelvic reconstruction after piecemeal resection of sacral giant cell tumors, with satisfactory preservation of bilateral S1–S3 nerve roots and long‐term implant stability, supporting the feasibility of personalized 3D‐printed prostheses in complex sacral defect reconstruction. The key to translating preoperative planning into accurate intraoperative execution lies in the use of patient‐specific 3D‐printed guiding templates. Designed via CAD simulations, these customized templates provide precise orientation for tumor resection and internal fixation. In the 3D‐ printing group, the templates enabled accurate placement of pedicle and iliac screws, thereby reducing the need for intraoperative fluoroscopy. Vissarionov et al. [5] confirmed that 3D‐printed guiding templates significantly improve screw placement accuracy in the thoracolumbar spine compared to freehand techniques. These findings suggest that 3D‐printed guiding templates not only enhance the precision of tumor margin resection but also significantly improve the accuracy of screw placement and internal fixation, ultimately improving surgical efficiency and clinical outcomes.

### 4.3 Efficiency Improvements From Preoperative Simulation

The simulation‐driven workflow fundamentally shifted the surgical paradigm from intraoperative improvisation to preoperative execution. By preemptively defining optimal screw trajectories and resection margins, the 3D templates minimized the need for repeated fluoroscopic verification, directly translating to shortened operative durations. Crucially, this technical precision extends beyond bone resection to vital soft tissue preservation. The superior hemostatic control observed in the 3D‐printing group was achieved through a synergistic “dual‐protection” mechanism. First, the precision‐guided templates facilitated strategic navigation around the presacral venous plexus, effectively avoiding iatrogenic vascular injury. Second, this anatomical precision worked in tandem with preoperative embolization to achieve hemorrhage control that surpassed traditional benchmarks, such as the anterior spinal column fixation (ASCF) technique reported by Bederman (4081 vs. 6650 mL) [20]. This intraoperative stability provides the essential foundation for accelerated postoperative recovery. The reduction in surgical trauma and the enhanced protection of sacral nerves allow for the earlier initiation of rehabilitation protocols, such as bedside sitting and protected standing. Such progress directly aligns with the principles of enhanced recovery after surgery (ERAS), ultimately explaining the significant reduction in hospitalization duration observed in our 3D‐printing cohort.

From a practical surgical perspective, several important strengths and technical considerations emerged from our experience. This template‐guided workflow enabled accurate translation of preoperative planning into intraoperative execution, reduced dependence on repeated fluoroscopic verification, improved osteotomy reproducibility, and facilitated individualized reconstruction in anatomically complex sacral tumors. In addition, patient‐specific guides enhanced multidisciplinary surgical coordination and procedural consistency.

The practicality of this technique should be interpreted within its appropriate clinical context. Owing to the approximately 1‐week lead time required for image processing, CAD planning, and template fabrication, this workflow is best suited for planned oncologic surgeries involving complex sacral tumors requiring en bloc resection rather than emergency procedures.

Several technical pitfalls should also be recognized. Accurate template placement depends on adequate exposure of predefined bony landmarks and meticulous soft‐tissue clearance. Furthermore, successful implementation requires a learning curve in CAD planning and guide verification. Based on our experience, repeated virtual simulation and strict intraoperative verification are essential for maximizing the benefits of this technology.

### 4.4 Clinical Implications and Limitations

From a clinical and study design perspective, the present study demonstrates several important advantages of integrating CAD‐assisted planning and patient‐specific guiding templates into complex sacral tumor surgery. The observed reductions in operative time, blood loss, fluoroscopic exposure, and screw‐placement deviation suggest that this workflow may significantly improve the reproducibility and accuracy of technically demanding procedures, which serves as a key highlight of our framework. Moreover, by translating preoperative virtual planning into intraoperative execution, the proposed strategy provides a practical framework for individualized surgical reconstruction.

However, these findings and advantages should be interpreted in light of several important limitations and disadvantages inherent to this retrospective cohort. First, the retrospective single‐center design and limited sample size (*n* = 19) inevitably introduce selection bias and limit generalizability. Our cohort included only operable Enneking IIb–III tumors involving S1–S3, excluding advanced inoperable lesions. Thus, our conclusions primarily apply to locally advanced but resectable sacral tumors.

Additionally, group allocation followed the chronological adoption of 3D‐printing technology (2015 as the cutoff), which may have led to more complex cases in the 3D‐printing group. Nonetheless, improvements in fluoroscopy frequency (4.2 ± 0.79 vs. 10.0 ± 1.58, *p* < 0.001) confirm the precision benefit of 3D guidance.

Second, patient heterogeneity may have affected outcome interpretation, as the cohort included multiple histological subtypes with distinct biological behaviors. This variability may partly explain the variability in follow‐up events, warranting cautious interpretation of oncological outcomes. The cohort also included benign‐aggressive entities such as giant cell tumor of bone. Although intralesional curettage is commonly considered for giant cell tumor when feasible, en bloc resection may be indicated for selected axial lesions with extensive involvement or complex anatomy [21].

Detailed stratification of sacral nerve root management and adjacent visceral resection was not systematically analyzed in this retrospective cohort, which represents an important limitation given their impact on functional outcomes. Future studies with larger sample sizes are warranted to incorporate nerve preservation and organ involvement into outcome assessment.

Resection margins were planned according to oncologic principles and individualized based on tumor extent; due to the inclusion criteria of this cohort, even benign‐aggressive lesions such as giant cell tumor involved extensive upper sacral regions, and therefore an en bloc resection framework was applied.

Third, subtle differences in tumor extent and neurovascular involvement within the same Enneking/Guo stage could introduce bias. Nevertheless, 3D‐printed guides helped standardize osteotomy planes and reduce variability between cases.

Fourth, while the 15‐year study period (2006–2020) may have introduced temporal confounders due to advances in imaging and surgical care, the unequal distribution of cases between groups over time may have affected the comparison and introduced potential bias.

Regarding osteotomy accuracy assessment, quantitative 3D coregistration between preoperative planning and postoperative CT models with millimeter‐level positional deviation measurement was not performed. This was primarily limited by the retrospective nature of imaging acquisition and extensive metal artifacts from spinopelvic fixation constructs. Consequently, angular deviation was used as a practical indicator to reflect the consistency between planned and executed osteotomy planes. Future prospective studies incorporating standardized imaging protocols may enable more comprehensive spatial accuracy evaluation.

We did not perform dedicated bench testing to quantify printing tolerances or stiffness‐related deformation of resin guides; therefore, material‐related manufacturing variability cannot be excluded.

Time and cost considerations also represent practical limitations. In our workflow, guide fabrication required approximately 1 week of lead time and an estimated direct cost of ~6000 RMB per case, which may restrict routine use in urgent settings. Nevertheless, prior economic analyses suggest that 3D‐printed surgical guides may reduce operating room time and partially offset fabrication‐related costs, particularly for technically demanding procedures.

Furthermore, patients were grouped according to their informed preference under comparable baseline conditions, which could still introduce selection bias. Future randomized multicenter studies are warranted to minimize such effects.

Finally, the median follow‐up of 31.5 months is insufficient to evaluate long‐term osseointegration and oncologic outcomes. Extended follow‐up is needed to validate the durability of 3D‐printed reconstructions.

Despite these limitations, our findings clearly demonstrate that 3D‐printed guiding templates improve preoperative planning, intraoperative precision, and surgical efficiency in complex sacral tumor resections. Future multicenter, prospective studies with larger cohorts and stratified analyses are needed to confirm these advantages and explore their long‐term clinical impact.

## 5 Conclusion

This study suggests that integrating CAD with patient‐specific 3D‐printed guiding templates can improve the technical precision and operative efficiency of sacral tumor resection. By facilitating preoperative simulation and individualized osteotomy planning, this approach may reduce intraoperative adjustments, optimize implant positioning, and simplify the surgical workflow. Compared with conventional freehand techniques, template assistance was associated with shorter operative time, fewer intraoperative fluoroscopy exposures, and improved osteotomy accuracy. While current 3D‐printed technologies offer clear technical advantages for sacral reconstruction, further research is warranted to optimize material properties and osseointegration of 3D‐printed implants. Future integration of template‐based planning with real‐time navigation and customized surface treatments may further improve long‐term outcomes. Ultimately, this technology standardizes complex sacral resections, making them more reproducible and safer.

## Author Contributions

**Yinghua He:** formal analysis, methodology, visualization, writing – review and editing. **Yansong Liu:** data curation, formal analysis, methodology, writing – original draft, writing – review and editing, visualization. **Zhongbiao Liang:** writing – review and editing, visualization. **Qiang Tu:** conceptualization, data curation, funding acquisition, methodology, writing – review and editing, project administration, visualization. **Hu Chen:** visualization, writing – review and editing, methodology. **Ningling Xie:** visualization, writing – review and editing. **Renxuan Tu:** writing – review and editing, visualization. **Huanwen Ding:** conceptualization, data curation, visualization, writing – review and editing.

## Funding

This study was financially supported by the Guangzhou Municipal Science and Technology Bureau under the Guangzhou Science and Technology Program Project (Grant No. 2025A03J3257).

## Ethics Statement

This retrospective study was approved by the Ethics Committee of the General Hospital of Southern Theater Command (Approval No.: NZLLKZ2025137).

## Conflicts of Interest

The authors declare no conflicts of interest.

## Supporting information


**Figure S1:** Additional preoperative simulation and intraoperative workflow details for the representative case presented in Figure 6.

## Data Availability

Research data are not shared.
