# Supplementary material for: Application of Three‐Dimensionally Printed Surgical Guides in Precise Sacral Tumor Excision and Defect Reconstruction
Source: Orthop Surg. 2026 Jul 26:10.1111/os.70383. Online ahead of print. doi: 10.1111/os.70383 (PMC13402848; doi:10.1111/os.70383)
Supplement: Supplementary file 1 — Figure S1: Additional preoperative simulation and intraoperative workflow details for the representative case presented in Figure 6. [file OS-9999-0-s001.pptx]

## Slide 1
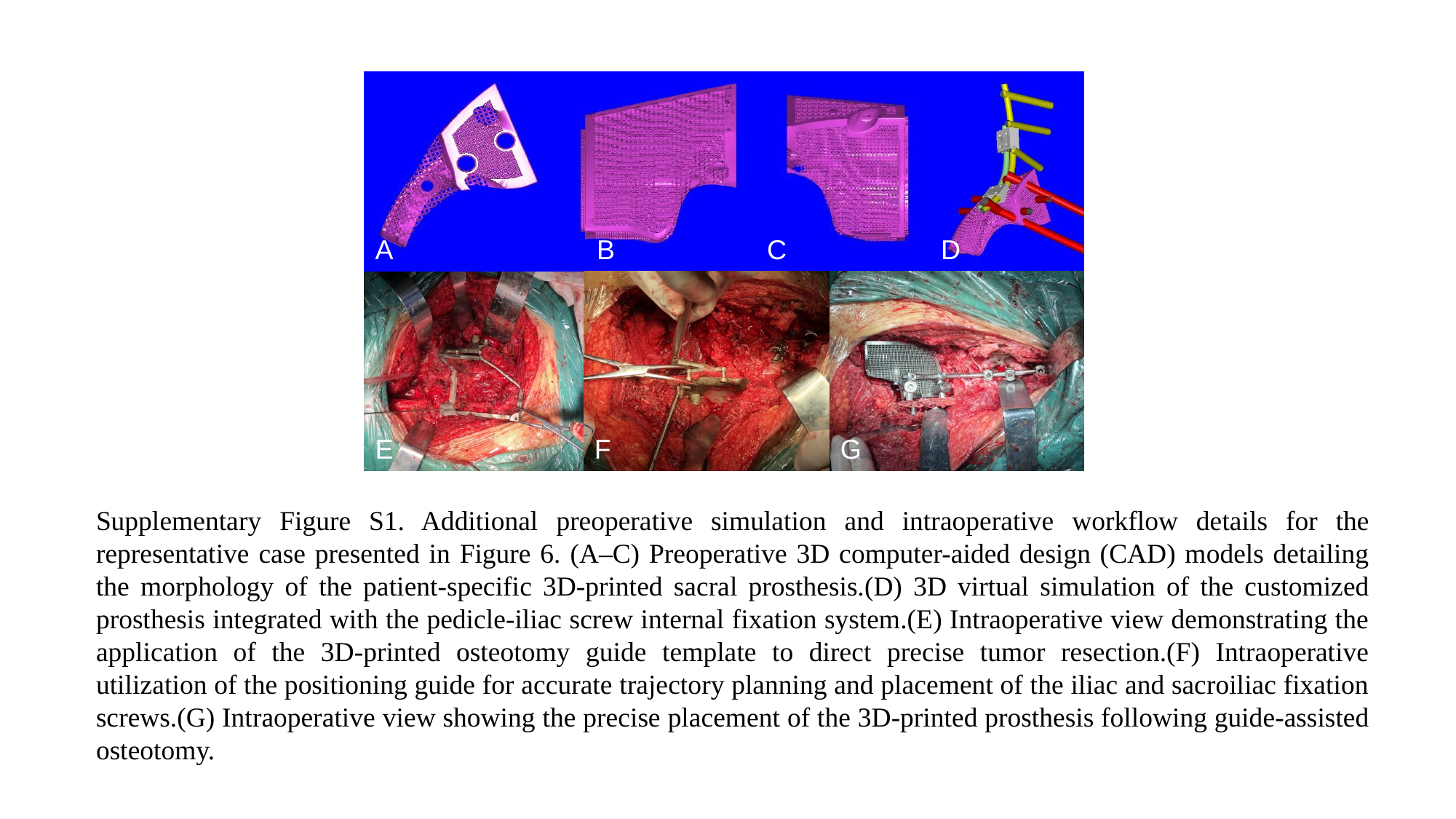

A
B
C
D
F
G
E
Supplementary Figure S1. Additional preoperative simulation and intraoperative workflow details for the representative case presented in Figure 6. (A–C) Preoperative 3D computer-aided design (CAD) models detailing the morphology of the patient-specific 3D-printed sacral prosthesis.(D) 3D virtual simulation of the customized prosthesis integrated with the pedicle-iliac screw internal fixation system.(E) Intraoperative view demonstrating the application of the 3D-printed osteotomy guide template to direct precise tumor resection.(F) Intraoperative utilization of the positioning guide for accurate trajectory planning and placement of the iliac and sacroiliac fixation screws.(G) Intraoperative view showing the precise placement of the 3D-printed prosthesis following guide-assisted osteotomy.
